# The implementation of online and offline hybrid weight management approach for pregnant women based on the Fogg behavior model in Hainan, China: a pilot randomized controlled trial

**DOI:** 10.1186/s12884-024-06699-2

**Published:** 2024-07-30

**Authors:** Linjie Wang, Lanli Zou, Huanying Yi, Tong Li, Rong Zhou, Jing Yang, Jia Wang, Caihong Zhang, Honghua Guo

**Affiliations:** International Nursing School, Hainan Medical University, Longhua District, No.3 Xueyuan Road, Haikou, 571199 Hainan Province China

**Keywords:** Gestational weight gain, Weight management, WeChat, Pregnant women, Randomized trial

## Abstract

**Objective:**

This study aimed at evaluating the effects of online and offline hybrid weight management approach based on the Fogg behavior model on total gestational weight gain and perinatal outcomes.

**Methods:**

Pregnant women in Hainan, the southernmost province of China, were recruited into a randomized controlled trial, which was designed to develop a WeChat platform for pregnancy weight management, and implement individualized and continuous pregnancy weight management services for pregnant women under the guidance of the Fogg behavior model. All pregnant women participating in the study were included in the full analysis set (FAS) for analysis. The pregnant women who completed the intervention and provided all outcome indicators were included in the per protocol set (PPS) for outcome evaluation.

**Results:**

Fifty-eight pregnant women were included in FAS analysis, and 52 pregnant women were finally included in PPS analysis. There was no statistically significant difference (*P* > 0.05) between the two groups at baseline. The gestational weight gain of the intervention group was significantly lower than that of the control group (*P* < 0.05). In the control group, the rate of appropriate weight gain during pregnancy was 48.26%, the rate of appropriate weight gain during pregnancy was 93.30% in the intervention group, with a statistically significant difference (*P* < 0.05). In the delivery outcomes, the cesarean section rate in the intervention group was significantly lower than that in the control group, and the differences were statistically significant (*P* < 0.05). The incidence of gestational diabetes mellitus and gestational hypertension in the intervention group was lower than those in the control group, and the differences were statistically significant (*P* < 0.05). The neonatal weight and incidence of macrosomia of the intervention group were lower than that of the control group, and the difference was statistically significant (*P* < 0.05).

**Conclusions:**

This study combined the individualized and continuous pregnancy weight management of the online WeChat platform and offline consultation based on the Fogg behavior model, showing great potential in improving maternal and infant outcomes.

**Trial registration:**

The study was registered with www.chictr.org.cn/index.aspx, Chinese Clinical Trial Registry (ChiCTR2200066707, 2022–12-14, retrospectively registered).

## Background

Gestational weight gain (GWG) is associated with maternal and neonatal outcomes. Studies [1–3] show that abnormal GWG is the risk factor for gestational diabetes mellitus, pregnancy-induced hypertension, cesarean section, macrosomia, intrauterine growth restriction, preterm birth, and even perinatal death. Additionally, abnormal GWG has also been proven to have a significantly high relation to postpartum depression [4]. In America, 48.6% of women have excessive gestational weight gain (EGWG), and only 31.5% of women have optimal weight gain during pregnancy [3]. In some low- and middle-income countries, these numbers are 15%, and 10% [5]. China also faces the dilemma that there are 50.1% women with EGWG, and only 32.6% women within a normal range [6]. In addition, a previous study shows that although more than half of the investigated women place a high priority on GWG, only 27.6% of them master the correct recommended GWG [7]. Therefore, how to carry out effective weight management and promote pregnant women to keep an appropriate GWG is now a great public health concern in the world.

Common pregnancy weight management involves face-to-face group sessions for health education and individual counseling with doctors, dietitians, or midwives [8]. These interventions advocating continuous individualized diet, exercise intervention services, and weight gain monitoring have confirmed the role they played in reducing childbirth complications and weight control [9, 10]. In China, weight management measures have gradually changed from collective weight management such as organizing lectures and watching videos to providing counseling services in individual health education such as targeted health education, lifestyle guidance, and weight monitoring based on the individual weight during pregnancy [11–13]. These personalized methods have achieved good results in controlling EGWG and improving delivery outcomes, but they pose a severe challenge to the current situation of the lack of human resources nurses and midwives in China [14]. In addition, most of the studies start interventions in mid-to-late pregnancy and the effects of interventions on pregnant women in early pregnancy need to be further validated. The abnormal GWG in the early and middle trimesters of pregnancy may be associated with the risk of gestational diabetes and hypertensive disorder of pregnancy [15, 16], so the earlier the intervention is implemented, the more beneficial it is to improve the initiative of pregnant women to manage their gestational weight and form health behavioral habits, which can effectively achieve the appropriate GWG [17, 18]. In the studies that started intervention late in pregnancy, some women were often close to or above the standard weight, and further weight control did not have much effect. When the effect was little, women's anxiety increased, further affecting the effect of weight management behavior and delivery outcomes [19, 20]. Thus, a scientific and more resource-saving method needs to be verified to help Chinese women manage their weight gain during gestation.

Using mHealth to manage pregnancy weight to solve the problem of human resources has become one of the current hotspots. An increasing number of pregnant women have smartphones, and they have a certain learning ability so it is easy for them to accept new things. These characteristics make it possible to bring mHealth into pregnancy services. At present, mobile technologies have been developed maturely in perinatal health services, and mHealth apps are taking the place of traditional text message or email services [21–23]. Some studies of weight management using apps and electronic devices have demonstrated the effectiveness [24–29]. The logistics and acceptability of social platforms such as using Instagram to intervene with GWG have also come to the attention of some researchers [30]. The mHealth is advantageous for pregnancy weight management, but studies [31] have also found that app usage declines over time, and some pregnant women are skeptical of the information provided in the app. Mobile wearable devices are simple and portable, but the operating system is still in the development stage and the effect on normal pregnant women is yet to be explored. In addition, even pregnant women are thoroughly educated before mHealth-based interventions, women still need one-on-one follow-up for estimates on how to manage like eating and exercising correctly. Therefore, a highly used mobile platform combined with a one-to-one consultation could make a difference.

WeChat is a social networking app that has Chinese and English versions. The combined monthly active accounts are over 1.2 billion in the world [32], showing great potential in mHealth. People can send voice, video, pictures, and text rapidly through cell phones or tablets for instant communication, which breaks the limitation of interpersonal communication distance. As a newly emerged way of pregnancy management, this platform is also gradually playing an active role in maternity examinations and health education. It has also gained wide attention from medical professionals and pregnant women, most of whom have a positive attitude towards it [33]. A study [34] conducting maternal nutritional guidance through WeChat to manage GWG showed a great effect on improving maternal outcomes, such as maternal BMI, and the incidence of maternal complications. In China, WeChat has also been widely used in health management studies [35–37]. However, there are still few studies on normal pregnancy management, especially in weight management, which is worthy of more attention.

To make the intervention more scientific, a theory of Fogg behavior model was chosen as a guide. According to this theory, people must have three elements at the same time to produce action: motivation, ability, and trigger. Only high motivation and ability with some stimulation can lead to the target behavior, effective stimulation can increase motivation and ability, and even when motivation and ability are high, some stimulation is needed to motivate the target behavior. Motivation includes feelings (pleasure or pain), expectations (hope or fear), and a sense of belonging (the desire to be accepted by others). Ability refers to the cost of accomplishing something, including time, money, physical effort, mental effort, social pressure, and past habits. These can indirectly affect a person's ability to behave. Behavioral trigger refers to the external stimulus for a person to complete a behavior [38, 39]. In this study, under the guidance of the Fogg behavior model, following an extensive review of literature and consultation with experts, the intervention plan was devised based on distinct stages of pregnancy encompassing three parts of motivation, ability, and triggering on women's weight, and the form was a hybrid offline and online approach based on WeChat for early weight management.

Therefore, this study aimed to assess the feasibility and efficacy of a hybrid online and offline approach for early weight management by developing a WeChat platform based on Fogg behavior model. We hypothesized that women receiving such weight management would have less weight gain, have a higher appropriate growth rate, be less likely to have pregnancy complications, and have a lower rate of cesarean section and macrosomia than women receiving standard care.

## Methods

The study was a randomized controlled trial conducted in Hainan, the southernmost province of China. The study was approved by the Institutional Review Board of Hainan Medical University (No. HYLL-2020–015). All participants were fully informed of the purpose and methods of the trial and signed informed consent.

### Participants

Pregnant women who were registered in the Obstetrics Clinic of the First Affiliated Hospital of Hainan Medical University from June 2020 to August 2020 were recruited. The inclusion criteria were as follows: (1) Primigravida with a singleton pregnancy with normal fetal development confirmed by ultrasound at the first obstetric consultation; (2) 18 years old ≤ age < 35 years old; (3) Registered in the outpatient clinic of the Obstetrics Department of the First Affiliated Hospital of Hainan Medical College (12–14 weeks of pregnancy); (4) Willing and proficient in using a smartphone with WeChat function during pregnancy; (5) Volunteering to participate in this study; (6) Having normal comprehension and able to cooperate with this study. The exclusion criteria were as follows: (1) Fetal malformation, complicated with pregnancy complications and comorbidities, history of recurrent abortion, stillbirth, preeclampsia, and other obstetric abnormalities; (2) Familial obesity, hypertension, and diabetes; (3) Participating in the WeChat platform guided by other professionals. The pregnant women enrolled in the trial were excluded in the following circumstances: (1) Lost contact; (2) Failure to cooperate or complete the study; (3) Unwilling or not allowed to play mobile phones; (4) Voluntarily withdrawing from the study; (5) Fetal malformation, abortion or premature birth occurred during the follow-up period.

There are reasons for choosing the women with 12–14 weeks of pregnancy. Given the insufficiency of weight management interventions during the early trimester, as mentioned in the background, this study aims to investigate the efficacy of interventions for pregnant women in early pregnancy. Currently, maternal records in Hainan province primarily rely on hospitals due to limited regional medical resources and incomplete development of community health care for pregnant women. The majority of pregnant women opt for hospital-based establishment of pregnancy records between 12 to 14 weeks. Therefore, this study selects the intervention period of 12 to 14 weeks gestation, ensuring scientific rigor and reliability while aligning with the local context.

### Sample size calculation

The formula for estimating sample size was based on the two population sample rates hypothesis tests of a completely random design.

The appropriate body weight growth rates of the two samples were obtained by literature review that the weight growth rate control group was nearly 58% (P1 = 0.58), and the intervention group was nearly 85% (P2 = 0.85). A two-sided test was used in this study. It was calculated that n1 = n2 = 26. Considering the sample loss rate of 10%, each of the intervention group and control group needs at least 29 cases.

$${n}_{1}= {n}_{2}= \frac{{\left[{u}_{a/2} \sqrt{2\overline{p }\left(1-\overline{p }\right)}+{u}_{\beta }\sqrt{{p}_{1} \left(1-{p}_{1}\right)+ {p}_{2}\left(1-{p}_{2}\right)}\right]}^{2} }{{\left({p}_{1}-{p}_{2}\right)}^{2}}$$

### Randomization

**Fig. 1:**
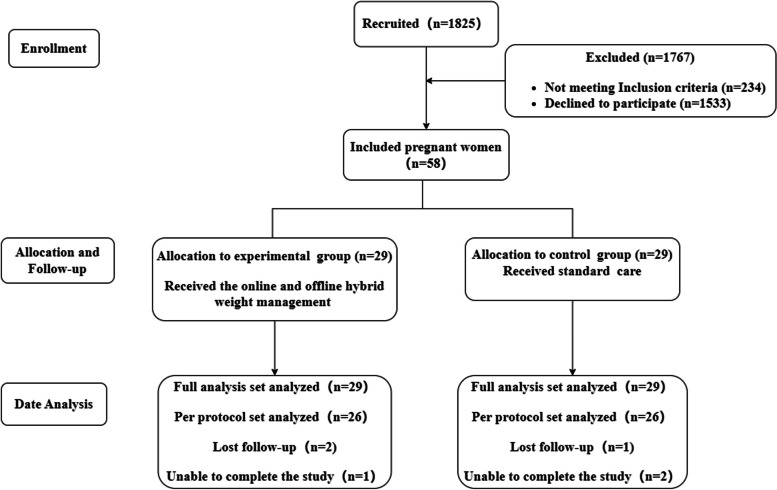
Study flow diagram

When pregnant women at 12–14 weeks of gestational age, a randomized controlled trial was conducted and randomly divided into two groups according to the random number table method, including 29 cases in the intervention group and 29 cases in the control group (allocation ratio = 1:1) (Fig. 1). The researchers themselves generated a table of random numbers through software, then put each grouping scheme into an opaque envelope with a code written outside, sealed it and handed it to another obstetrician. When the subjects entered the study, the researchers numbered the subjects. The obstetric staff then opened the numbered envelopes and grouped them.

### Setting

The study was conducted at a tertiary Class A hospital in Haikou, Hainan Province, China. Hainan is an independent island located in the southernmost part of China. Its GDP in 2020 was 553.239 billion yuan, and the per capita disposable income of permanent residents in the province was 27,904 yuan [40], which was relatively backward. There were 1310 births in 2020 in the hospital, and the average cesarean section rate was nearly 53%. The maternity clinic of the hospital had a special offline maternity school, and all the teaching content was developed and taught by midwives and obstetricians so that even the pregnant women included in the control group could still have the opportunity to learn regular pregnancy information according to their wills.

Although the study was conducted during the COVID-19 pandemic, according to the official statistics, on July 8, the cumulative number of confirmed infectious cases in Haikou was 39 [41]. After that, the Hainan Provincial Health Commission did not continue to report the number of new daily infections, and only one imported case was reported in October, indicating that the epidemic situation in Haikou was leveling off and people were returning to normal life. The impact on pregnant women was minimal, allowing the study to proceed.

### Standard care

In the control group, the interventions lasted from the beginning of inclusion to the end of delivery, and routine pregnancy health education was used. Pregnant women received regular prenatal examinations according to the recommendation [42] in the hospital and were taught maternity school courses during the examinations, which mainly included courses on preparation and coping skills for delivery, self-monitoring during pregnancy, prenatal diagnosis, exercise during pregnancy, guided delivery, postpartum rehabilitation, breastfeeding, newborn vaccination, and newborn bathing, etc. And the universal education of weight management was included in these routine courses. Pregnant women arranged the time and frequency of classes according to their needs and time. We distribute health education materials on pregnancy, childbirth, puerperium, breastfeeding, etc., and other general health education during pregnancy. Doctors and midwives usually pay attention to the weight of the pregnant woman and the fetus during late labor evaluation to determine whether a natural birth could be easily conducted.

### Online and offline hybrid weight management

The intervention group consisted of personalized and continuous pregnancy weight management via online and offline care.

#### Establishment of an intervention group

A pregnancy weight management intervention group was established, comprising of one obstetrician, one dietitian, two midwives, and one graduate student of midwifery. The obstetrician was responsible for outpatient prenatal examinations and result consultations; the dietitian provided nutrition consultation and guidance; the midwives were responsible for offline and online weight management consultations; while the graduate student applied for a WeChat public account, established WeChat groups, guided and maintained the use of the platform, as well as collected information from pregnant women.

#### Development of intervention measures

The intervention plan was primarily grounded in the Fogg behavioral model. Following an extensive review of literature and consultation with experts, the intervention plan was devised in four stages based on distinct stages of pregnancy, and each part contained the three elements of Fogg behavioral model (motivation, ability, and triggering) on pregnant women's weight. The use of elements of Fogg behavioral model was as follows:

1. **Motivation**: Through a combination of online and offline methods, tailored weight management goals are set for pregnant women to stimulate their motivation to achieve pregnancy weight control goals, and weight management guidance is provided for health education to improve pregnant women's motivation.
2. **Ability**: The primary objective of this component is to enhance the weight control capability and facilitate the adoption of weight control behaviors among pregnant women by reducing the difficulty and cost associated with such behaviors. It pushes nutrition and exercise knowledge of weight control through the public account and WeChat group. Additionally, the WeChat public account page features two sub-menus: "Latest Content" and "Previous Content," enabling pregnant women to swiftly access relevant information. Moreover, an advisory services sub-menu is provided, which includes contact details of the research team. Furthermore, the public account page allows for seamless communication with researchers via voice messages, text-based queries, expressions, and other means.
3. **Triggering**: Triggering is an intervention measure implemented by the research team to facilitate pregnant women in carrying out crucial milestones of weight management, encompassing three key strategies: stimulation, assistance, and signaling. Stimulation involves motivating pregnant women with the capacity for weight management during pregnancy through imparting health education knowledge and presenting cases of successful weight control. Assistance refers to providing comprehensive guidance both online and offline when pregnant women possess the willingness and motivation to manage their weight but lack the necessary skills or clarity regarding appropriate dietary choices and exercise routines. Signal entails reminding pregnant women about the significance and methods of weight control via online health education resources as well as face-to-face counseling sessions, while also keeping them informed about their current weight management progress through regular tracking.

The more detailed intervention measures proposed by the WeChat platform based on Fogg behavior model are available in Table 1.

**Table 1:** Intervention measures proposed by WeChat platform based on Fogg behavior model

Intervention parts	12–14 weeks of pregnancy	15–27 weeks of pregnancy	28–36 weeks of pregnancy	37 weeks of pregnancy to delivery
Motivation	1.Face-to-face counseling services were provided to pregnant women enrolled in the study 2.Pregnant women were invited to set individualized weight goals together 3.After setting the overall target of GWG, the weight gain targets of pregnant women at each stage of pregnancy were set combined with the results of ultrasound examination, uterine height and abdominal circumference, and fetal development 4.Emphasizing the importance of weight control during pregnancy and the relationship between weight gain during pregnancy and delivery mode and neonatal growth and development 5.Assessing the support of family members, encouraging their participation and support to enhance pregnant women's confidence and motivation for weight control	1.WeChat friends supervised each other, and midwives monitored the GWG of pregnant women 2.WeChat public number pushes pregnancy health education knowledge to strengthen pregnant women's awareness of weight control 3.Pregnant women exchanged their weight control experiences and goals through WeChat groups 4.Combined with the weight control goals, WeChat red envelopes were used to praise pregnant women with good weight control 5.Offline consulting services were provided once every 4 weeks. Each time, 20–30 min of face-to-face communication were provided to make a staged assessment of the GWG and guidance of pregnant women continue to assess and encourage family participation	1.On the basis of the measures in the previous stage, the offline consultation services were changed to once every two weeks. 2.More focus was given to the pregnant women with gestational diabetes and gestational hypertension, and the importances of weight control were emphasized.	1.On the basis of the previous stage, the importance of weight control in this stage for childbirth was emphasized to reduce the complications during childbirth and stimulate the motivation of pregnant women for weight control. 2.The offline consultation services were changed to once a week.
Ability	1.Midwives added pregnant women's WeChat friends and invited pregnant women to enter a specific WeChat group. Pregnant women were encouraged to introduce themselves in the WeChat group to get familiar with each other 2.Every pregnant woman was asked to follow the "expectant mother weight management WeChat public account"	Pregnant women were encouraged to ask questions and consult online in the form of one-to-one or WeChat group. The weight control of pregnant women and the number of WeChat exercise steps were urged and monitored through WeChat. Every week through the WeChat public account to publish the relevant knowledge of weight control in the middle and late pregnancy, to indirectly reduce the cost of health education and improve the weight control ability of pregnant women.		
Trigger	1.The first offline diet assessment, exercise assessment and lifestyle assessment were carried out. Combined with the dietary guidelines for Chinese residents, face-to-face nutrition guidance was given to the pregnant women in view of their diet problems and their pre-pregnancy BMI values 2.Appropriate exercise types were selected combined with the exercise guidelines for pregnancy issued by the American College of Obstetricians and Gynecologists and the subjective feelings of pregnant women. Pregnant women were guided to develop a healthy lifestyle.	1.Stimulation: The current stage of pregnancy weight management health education knowledge and weight control cases are released weekly through WeChat to stimulate pregnant women's motivation 2.Assistance: The midwives provided detailed nutrition, exercise, and lifestyle guidance during pregnancy every four weeks to provide assistance 3.Signal: Midwives reminded pregnant women of the importance and ways of weight control through WeChat, assessed the weight of pregnant women offline, and reminded pregnant women of the existing problems and the precautions in the next stage, playing the role of signal reminder.	1.Stimulation: Based on the measures of previous stage, pregnant women diagnosed with gestational diabetes mellitus and gestational hypertension were emphasized the dangers of the disease to stimulate their motivation 2.Assistance: Midwives provided offline assistance and guidance on detailed pregnancy nutrition, exercise and lifestyle once every two weeks, and special dietary guidance was given to the pregnant women diagnosed with gestational diabetes and gestational hypertension 3.Signal: Based on the measures of previous stage, guidance was provided for pregnant women with gestational complications.	1.Stimulation: Based on the measures of previous stage, the precautions during childbirth and the connection between childbirth and weight control were added 2.Assistance: Midwives provided assistance and detailed guidance once a week on nutrition, exercise, lifestyle and labor recognition during the third trimester to help pregnant women increase the confidence of natural childbirth 3.Signal: Based on the measures of previous stage, the implement of weight management was reminded and the confidence of natural childbirth was improved to prevent complications during childbirth.

### Outcomes

In this study, a general demographic information questionnaire was used to investigate the general situation of patients, including age, education level, family per capita monthly income, body mass index (BMI) before pregnancy, gestational age, gestational age in childbirth, etc. By comparing the main health education consultation problems in different stages, the health education consultation problems in the first three stages in the WeChat group and the face-to-face service in the outpatient department were statistically analyzed in the two groups of pregnant women. The main evaluation indexes included: the appropriate rate of pregnancy weight gain, weight gain during pregnancy, pregnancy complications, cesarean section rate and the incidence of macrosomia.

### Statistical analyses

IBM-SPSS24.0 software was used for data analysis. The measurement data in this study were presented as mean ± standard deviation($\overline{\boldsymbol{x}}$ ± s). T-test was used for those meeting the normal distribution, and Mann–Whitney U-test was used for those not meeting the normal distribution presenting as median (quartile). Chi-square test was used for comparison of the enumeration data. *P* < 0.05 was considered as statistically significant. Meanwhile, the statistical analysis of this study followed the principle of intention-to-treat (ITT) analysis. All pregnant women participating in the study were included in the full analysis set (FAS) for analysis. And the pregnant women who completed the intervention and provided all outcome indicators were included in the per protocol set (PPS) for outcome evaluation. The mean imputation method was used for missing data.

## Results

From June 2020 to August 2020, 58 pregnant women were initially enrolled in the obstetrics department of the First Affiliated Hospital of Hainan Medical University. During the research, a total of 6 pregnant women fell off, including 3 cases who lost follow-up and 3 cases who were excluded because they went to other cities and could not complete the follow-up. Therefore, 58 pregnant women were included in the FAS analysis, and 52 pregnant women were finally included in the PPS analysis. Whether it was FAS analysis or PPS analysis, there was no statistically significant difference (*P* > 0.05) between the two groups in terms of age, gestational age, pre-pregnancy BMI, gestational age, education level, per capita monthly income of the family and other general information, showing a balance and comparability. See Tables 2 and 3.

**Table 2:** Comparison of general demographic data between the two groups of subjects (PPS analysis)

Item	Control group *N* = 26	Intervention group *N* = 26	*t/χ*^*2*^*/ Z*	*P*
**Age (years) (**$\overline{\boldsymbol{x}}$ **± s)**	29.15 ± 3.45	29.96 ± 3.07	-0.892	0.376
**Pre-pregnancy BMI (**$\overline{\boldsymbol{x}}$ **± s)**	21.44 ± 3.04	21.1 ± 2.27	-0.465	0.644
**Gestational age at delivery (week) M (P25,P75)**	39(38,39)	39(38,39)	-1.089	0.276
**Education level**			0.926	0.629
Junior college or above (n, %)	13(50.0)	15(57.69)		
High school or technical secondary school (n, %)	8(30.77)	5(19.23)		
Junior high and below (n, %)	5(19.23)	6(23.08)		
**Monthly household income**			0.340	0.560
> 3,000 RMB (n, %)	16(61.54)	18(69.23)		
≤ 3000 RMB (n, %)	10(38.46)	8(30.77)		
**Conception mode**			0.1448	0.701
Natural (n, %)	21(80.77)	23(88.46)		
Assisted reproductive (n, %)	5(19.23)	3(11.54)

**Table 3:** Comparison of general demographic data between the two groups of subjects (FAS analysis)

Item	Control group *N* = 29	Intervention group *N* = 29	*t/χ*^*2*^*/ Z*	*P*
**Age (years) (**$\overline{\boldsymbol{x}}$ **± s)**	29.13 ± 3.66	30.00 ± 3.64	0.899	0.373
**Pre-pregnancy BMI (**$\overline{\boldsymbol{x}}$ **± s)**	21.50 ± 2.88	21.19 ± 2.18	-0.462	0.646
**Gestational age at delivery (week) M (P25,P75)**	38.4 (38,39)	39 (38,39)	-0.187	0852
**Education level**			1.056	0.590
Junior college or above (n, %)	16 (55.17)	17 (58.62)		
High school or technical secondary school (n, %)	8 (27.59)	5 (17.24)		
Junior high and below (n, %)	5 (17.24)	7 (24.14)		
**Monthly household income**			0.305	0.581
> 3,000 RMB (n, %)	18 (62.07)	20 (68.97)		
≤ 3000 RMB (n, %)	11 (37.93)	9 (31.03)		
**Conception mode**			0.145	0.701
Natural (n, %)	24 (82.76)	26 (89.66)		
Assisted reproductive (n, %)	5 (17.24)	3 (10.34)		

### GWG

The GWG of the intervention group was significantly lower than that of the control group, and the difference was statistically significant (*P* < 0.05). In the control group, there were 11 cases of appropriate weight gain during pregnancy, and the rate of appropriate weight gain during pregnancy was 48.28%, while in the intervention group, there were 24 cases of appropriate weight gain during pregnancy, and the rate of appropriate weight gain during pregnancy was 93.10%. When comparing the pregnancy weight gain appropriateness rate of the two groups, the pregnancy weight gain appropriateness of the intervention group was significantly better than that of the control group, and the difference was statistically significant (*P* < 0.05), and all the results of FAS analysis were consistent with those of PPS analysis. The specific results are shown in Tables 4 and 5.

**Table 4:** Comparison of pregnancy weight gain between the two groups (PPS analysis)

	**Control group** ***N* = 26**	**Intervention** ***N* = 26**	***χ***^***2***^***/ Z***	***P***
Weight gain during pregnancy (kg) M (P25,P75)	15 (14, 17)	13 (12, 14)	-13.695	0.000
Appropriate rate of GWG (n, %)	11 (42.31)	24 (92.31)	12.585	0.000

**Table 5:** Comparison of pregnancy weight gain between the two groups (FAS analysis)

	**Control group** ***N* = 29**	**Intervention** ***N* = 29**	***χ***^***2***^***/t***	***P***
Weight gain during pregnancy (kg) **(**$\overline{\boldsymbol{x}}$ **± s)**	15.31 ± 3.26	13.28 ± 1.37	-3.080	0.003
Appropriate rate of GWG (n, %)	14 (48.28)	27 (93.10)	14.063	0.000

### Delivery outcomes

In the delivery outcomes, the cesarean section rate in` the intervention group was significantly lower than that in the control group, and the differences were statistically significant (*P* < 0.05). Meanwhile, the incidence of complications of gestational diabetes mellitus and gestational hypertension in the intervention group was lower than those in the control group, and the differences were statistically significant (*P* < 0.05). In terms of neonatal delivery outcomes, the neonatal weight of the intervention group was significantly lower than that of the control group, and the difference was statistically significant *(P* < 0.05). The incidence of macrosomia in the intervention group was significantly lower than that in the control group, and the difference was statistically significant (*P* < 0.05), and all the results of the FAS analysis were consistent with those of the PPS analysis. (See Tables 6 and 7).

**Table 6:** Effect of different interventions on maternal and delivery outcomes (PPS analysis)

	**Control group** *N*= 26	**Intervention group** *N*= 26	*χ*^*2*^*/ Z*	*P*
**Categorical outcomes**				
Cesarean section (n, %)	18 (69.23)	9 (34.62)	4.930	0.026
Gestational diabetes mellitus (n, %)	9 (34.62)	3 (11.54)	3.900	0.048
Gestational hypertension (n, %)	8 (30.77)	1 (3.85)	4.837	0.028
Macrosomia (n, %)	6 (23.08)	0 (0)	4.710	0.030
**Continuous outcomes**				
Newborn weight (g), M (P25,P75)	3420 (3050,4000)	3050 (2870,3300)	144.203	0.000

**Table 7:** Effect of different interventions on maternal and delivery outcomes (FAS analysis)

	**Control group** *N*= 29	**Intervention group** *N*= 29	*χ*^*2*^*/ t*	*P*
**Categorical outcomes**				
Cesarean section (n, %)	21 (72.41)	12 (41.38)	5.695	0.017
Gestational diabetes mellitus (n, %)	10 (34.48)	3 (10.34)	4.858	0.028
Gestational hypertension (n, %)	8 (27.59)	1 (3.45)	4.735	0.030
Macrosomia (n, %)	6 (20.69)	0 (0)	4.647	0.031
**Continuous outcomes**				
Newborn weight (g), **(**$\overline{\boldsymbol{x}}$ **± s)**	3411.74 ± 351.81	3095.19 ± 203.33	-4.195	0.000

## Discussion

This study aimed to evaluate the effects of online and offline hybrid weight management approach on total gestational weight gain and perinatal outcomes. Consistent with the hypothesis, online and offline hybrid weight management approach based on Fogg behavior model could significantly improve the appropriate rate of weight gain during pregnancy, and promote maternal and neonatal outcomes.

The results showed that the study participants in the intervention group gained less weight during pregnancy and the appropriate rate of weight gain during pregnancy of ITT analysis was 93.10%, which is similar to the results of the offline-based individualized lifestyle study conducted by Luo et al. [43]. But, in Luo's study, the appropriate rate of weight gain during pregnancy in the intervention group was 86%, which was lower than that in this study. This might be related to the full integration of the WeChat platform and the joint role of midwives and information technology under the guidance of Fogg behavior model. By maintaining close contact with pregnant women over time, their compliance increased and they were more likely to cooperate with the midwives. According to Fogg behavior model, this study provided pregnant women with the knowledge and ability of weight management through regular health knowledge and instant online answers, which promoted the change of weight management behavior, which was also similar to the results of some studies on weight management based on WeChat [44, 45]. At the same time, the results of this study were different from those of a systematic review [46]. The systematic review showed that six studies found no significant difference in the effect of maternal weight gain compared to controls of pregnant women. This might be related to the lack of completeness of the design elements of the interventions included in the study. Most of the included studies used limited regular text messages to monitor pregnant women's weight management and get feedback and lacked measures to improve pregnant women's weight management motivation and to increase external stimulation compared with our study. Based on Fogg behavior model, this study focused on immediate feedback and information sharing, improved weight management ability in pregnant women, and focused on intervention of motivation and triggering behaviors, which might have contributed to the different results. Through the supervision of midwives, the frequency of pregnant women's health knowledge learning can be enhanced. Additionally, face-to-face encouragement and WeChat red envelopes would be provided to those who achieve weight control targets to promote motivation. Case sharing and personalized guidance can trigger behavioral changes. All these interventions may have contributed to the observed results.

Most studies have focused on weight management in pre-pregnancy overweight or obese pregnant women, but pregnant women with a normal pre-pregnancy BMI are still at risk for suboptimal weight gain during pregnancy and subsequent other pregnancy complications and adverse birth outcomes [3]. This study included pregnant women with a normal range of pre-pregnancy BMI for weight management, which also proved to be effective. The intervention group had a significantly higher proportion of appropriate weight gain than the control group and also showed better outcomes in terms of pregnancy complications and birth outcomes. The reason might be that this study promoted the behavioral change of weight control during pregnancy by controlling factors affecting weight gain during pregnancy under the guidance of Fogg behavior model. Study [47] showed that factors affecting weight gain during pregnancy include a lack of understanding of EGWG and its risk during pregnancy, a lack of access to adequate, correct and scientific nutritional information, awareness of the benefits of physical activity during pregnancy, and lacking of self-efficacy in weight management during pregnancy [39]. In the course of this study, the researchers fully considered the causes associated with weight gain during pregnancy and achieved the goal of promoting behavior by improving the three parts of the Fogg behavior model. Pregnant women were not only treated as knowledge recipients, but were encouraged to discuss pregnancy weight management plans together. Through the suggestion of goal control, pregnant women participated in the self-management of pregnancy weight. Their endogenous motivation for weight control was stimulated, thus promoting the improvement of poor diet, exercise, and lifestyle, and the rate of the pregnancy weight in the appropriate range. The achievement of the goal, in turn, increased pregnant women's confidence in weight management. As a result, pregnant women in the intervention group were actively involved in achieving their pregnancy weight gain goals. Jia et al. [48] combined Fogg behavior model and mHealth, effectively improving the executive ability and management ability of ordinary users in self-management. Therefore, future intervention studies should focus on enhancing the motivation and self-efficacy of weight management in pregnant women, which are the basis for enhancing self-management of weight.

At the same time, an early intervention that extends throughout pregnancy may be the reason that the outcome measures in the intervention group are different from those in the control group. Paying attention to weight management early in pregnancy is crucial. A study [15] showed that excessive GWG during early and mid-pregnancy is linked to a higher risk of GDM. Furthermore, in a study conducted, it was found that 77.3% of participants exceeded the upper limit recommended by the Institute of Medicine (IOM) for weekly weight gain after 20 weeks of pregnancy. Meanwhile, EGWG during the first trimester and subsequent weekly weight gain beyond the recommended limit, which is positively associated with the risk of hypertensive disorders of pregnancy [16]. These studies collectively emphasize the importance of early monitoring of weight gain. Additionally, a prospective randomized clinical trial involving overweight/obese Chinese women demonstrated that initiating regular and light exercise early on could effectively reduce GWG [17]. Thus, it is a positive trend that pregnant women start to pay attention to their own weight management at the end of the first trimester. In addition, with early attention and management, pregnant women can become more involved in their own health and pay attention to the health of their babies during pregnancy. It has the potential to prompt their self-regulatory control which might be a trigger of weight management. Meanwhile, it also reflected the importance of pregnant women to their own health and fetal health, which is helpful in improving pregnancy health awareness and behavior [49]. Therefore, pregnant women are encouraged to pay attention to their weight management as early as possible during pregnancy, starting from the early establishment of a pregnant woman's medical records.

The ITT analysis results of this study showed that the rate of cesarean section of the intervention group was lower than the control group (41.38% V.S. 72.41%). Similar to the findings of Silva-Jose et al. [50], their study started the intervention between 12 and 14 weeks of gestation, and significant differences were found in the mode of delivery. There was no macrosomia in the intervention group, which was similar to the results of Zhou [51]. In this study, weight management was conducted in early pregnancy to promote the change of weight control behavior of pregnant women, avoid excessive intake of fat, sugar, and calcium during pregnancy, and control maternal and fetal nutrition intake. On the one hand, this measure ensured moderate growth of pregnancy and fetal weight and reduced the occurrence of macrosomia [52, 53]. On the other hand, reasonable control of body weight, avoided excessive increase in adipose tissue and cholesterol deposition in the abdominal wall and myometrium, reducing the factors that hinder vaginal delivery. In addition, the incidence of gestational diabetes and hypertension in the intervention group was lower than that in the control group, possibly by increasing insulin sensitivity and reducing oxidative stress [54]. Study shows that intestinal probiotics may influence insulin resistance by regulating the expression of peroxisome proliferator-activated receptor [55]. The main outcomes of this study were weight gain during pregnancy and delivery outcomes, but there was still a lack of evaluation of laboratory data, such as changes in intestinal microorganisms. Therefore, future studies could consider discussing the relationship between changes in intestinal microorganisms and weight gain.

Although this study did not systematically collect pregnant women's feelings about weight management during pregnancy through qualitative research, the researchers felt pregnant women's favor and trust in this project during daily consultation and communication. Many pregnant women said that this weight control method hosted by professionals on the WeChat platform, could let them get the right knowledge, make them feel reliable, and relieve anxiety and confusion, similar to the result of a meta-analysis [46]. This approach made them more willing to take action and more confident in normal delivery, thus promoting a change in weight control behavior and avoiding the occurrence of non-indicative cesarean section in some aspects [47, 56]. This also indicated that the intervention based on the motivation, ability and trigger parts of Fogg behavior model was effective and acceptable to pregnant women. Many women also expressed satisfaction with their pregnancy figure, great interest in the type of clothing they were wearing, and anticipation of delivery and the birth of their baby. After delivery, many pregnant women hoped that our weight management could continue to the postpartum period and provide postpartum weight management services. This was consistent with the results of Holton's study [57], which found that pregnant women hope to change the current situation of lack of information provided by nursing service providers and hope to get more information and support on weight management during pregnancy and postpartum. In the future, how to continue to combine the Fogg behavior model for postpartum weight recovery intervention deserves further discussion.

The utilization of WeChat as an intervention method is indeed prevalent currently. However, the study was implemented in the context of the normalization of prevention and control measures for the COVID-19. Due to the shortage of medical resources, many healthcare institutions have implemented measures such as restricting the number of patients, postponing non-emergency procedures and diagnoses. Consequently, pregnant women encountered challenges in scheduling appointments and experienced delays in accessing timely medical advice and services. During the epidemic, the provision of health education and telemedicine services for pregnant women to ensure their health and safety was facilitated by the use of the WeChat platform. While there may no longer be a strict prevention and control system for the COVID-19 epidemic worldwide, the intervention measures of our study can still serve as a reference for areas with uneven distribution of medical resources, difficulties in seeking medical treatment, or economically underdeveloped regions. In addition, WeChat is the most used and frequently used communication tool in China, and almost everyone among adult women of childbearing age has downloaded it. The ease of access provided by WeChat may also increase the motivation of pregnant women to participate in health management, as there is no need for them to log into a specific website or download a new app to take part in the intervention [58]. Lin et al. developed a weight management program using the WeChat platform for employees with varying health risk levels in China. The results indicated that the intervention significantly reduced weight at the endpoint (*P* < 0.001), and after 6 months, 67% of the individuals had no additional weight gain [59]. In addition, another study demonstrated that WeChat-assisted dietary and exercise interventions can effectively reduce the occurrence of GDM and EGWG in overweight/obese pregnant women [35]. Therefore, WeChat platform-based health interventions for weight management achieved positive results, further confirming the potential of WeChat in promoting weight management for pregnant women.

Of course, the limitations of WeChat intervention should also be acknowledged. Because communication for pregnant women is not scheduled, midwives may be questioned around the clock, increasing the workload, which is a major obstacle to the current management of pregnancy led by many midwives. However, to overcome this obstacle and enhance the methods of WeChat usage, future research should consider incorporating advanced technologies such as Internet of Things (IOT) and decision support systems based on big data computation. By analyzing extensive health data, medical professionals can make scientific and accurate decisions while providing intelligent and personalized immediate adaptive interventions for pregnant women. For instance, Sarhaddi et al. [60] designed an IOT-based maternal health system that utilizes various data collectors to monitor maternal stress, sleep patterns, and physical activity, enabling comprehensive healthcare monitoring and support regardless of location. To address the gap in delivering high-quality prenatal care at the primary healthcare level in India, Mohan's team developed an electronic decision support system that automatically delivers a personalized and evidence-based prenatal care process tailored to each pregnant woman's specific needs [61]. Furthermore, future research should explore integrating the WeChat platform with big data analysis and artificial intelligence.

### Advantages and disadvantages

**Fig. 2:**
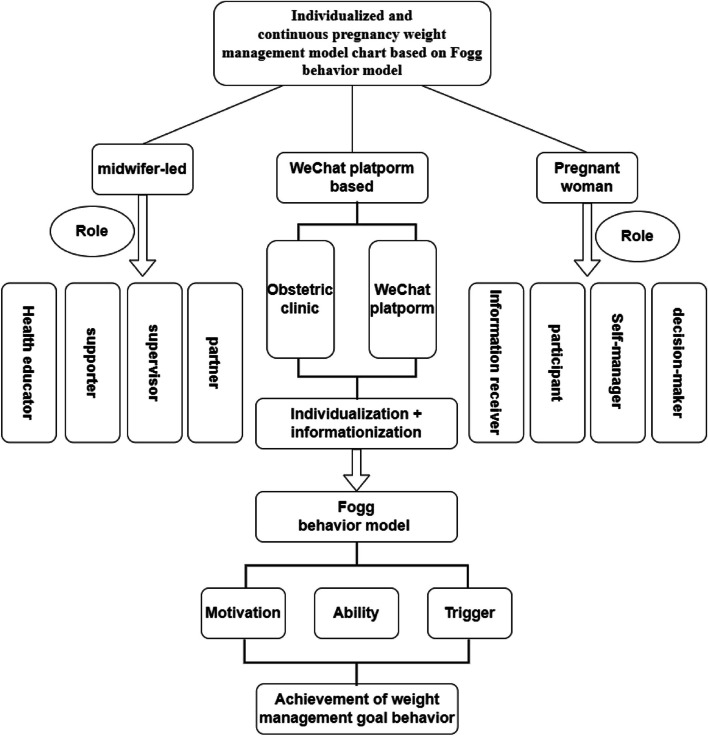
Individualized and continuous pregnancy weight management model chart based on the Fogg behavior model

As far as we know, this is one of the few projects in mainland China using the Fogg behavior model to explore the combination of online and offline hybrid pregnancy weight management throughout pregnancy. This study has developed a more comprehensive model of weight management during pregnancy that is worthy of clinical promotion (Fig. 2), which may play a role in promoting relatively weak midwifery services currently in China. At present, most of the practice forms led by midwives focus on mid- and late-pregnancy women, but lack systematic, individualized management for the whole pregnancy period [38]. The continuous management of pregnant women in this study shows the important role of midwives in weight management from early pregnancy.

For the shortcomings in this study, the small sample size of this study might have a bias in the conclusions. In addition, the observation indexes in this study only involved pregnancy weight, neonatal weight, mode of delivery, incidence of macrosomia, gestational diabetes, and incidence of gestational hypertension, and did not evaluate physiological, biochemical, and psychological indexes, which did not fully and adequately reflect the effect of weight management. At the same time, due to research limitations, this study was a midwife-led study in the process of conducting, but the current development of midwifery services in mainland China still suffers from a lack of manpower and promotion difficulties, which needs to be guaranteed from information technology level and policy perspective, and hospitals and obstetrics departments need to pay attention to maternal and fetal weight issues to improve the current situation.

## Conclusion

This study combined the individualized and continuous pregnancy weight management of the online WeChat platform and offline consultation based on Fogg behavior model, which reduced maternal weight, the incidence of macrosomia, cesarean section rate, and pregnancy complications, showing great potential in improving maternal and infant outcomes. In addition, it provided recommendations for pregnancy weight service guidelines, and formed a model that might be replicated nationally and internationally, especially in some regions having limited resources in midwifery and uneven distribution of medical resources. Future studies are needed to increase the sample size, improve the evaluation indicators, such as maternal self-efficacy, and pregnancy stress, and collect the postpartum weight of pregnant women and neonatal weight gain data and other long-term effects data. The psychological process of weight management in pregnant women can also be discussed to enrich the understanding of weight management in pregnant women. In the future, the weight management model of pregnant women with twin pregnancies can also be discussed. Meanwhile, the correlation between weight gain during pregnancy and intestinal microorganism and biochemical indicators will also be the future direction of further research. For the current situation of insufficient midwife manpower, the future application of big data to provide timely adaptive intervention for pregnant women may be the future research direction.

## Data Availability

The datasets used and/or analyzed during the current study are available from the corresponding author upon reasonable request.

## Abbreviations

GWG: Gestational weight gain
IOM: The Institution of Medicine
EGWG: Excessive gestational weight gain
BMI: Body-mass-index
ITT: Intention-to-treat
FAS: Full analysis set
PPS: Per protocol set
